# Polyphenols in the Fermentation Liquid of *Dendrobium candidum* Relieve Intestinal Inflammation in Zebrafish Through the Intestinal Microbiome-Mediated Immune Response

**DOI:** 10.3389/fimmu.2020.01542

**Published:** 2020-07-17

**Authors:** Xiaoyue Gong, Shuaiming Jiang, Haiyan Tian, Dong Xiang, Jiachao Zhang

**Affiliations:** ^1^College of Food Science and Engineering, Hainan University, Haikou, China; ^2^Key Laboratory of Food Nutrition and Functional Food in Hainan Province, Hainan University, Haikou, China

**Keywords:** intestinal inflammation, intestinal microbiota, *Dendrobium candidum*, short-chain fatty acid, gut mucosal barrier, fermentation

## Abstract

Previous studies of *Dendrobium candidum* (*D. candidum*), which is mainly distributed in tropical areas, have mainly focused on its functional polysaccharide; the effects of *D. candidum* polyphenols, the chemical composition of which may be improved by fermentation, have received limited attention, especially in *in vivo* models, which inevitably involve interactions with intestinal microorganisms. To address this challenge, metagenomic and metabolomic techniques, were applied, and immune factors and mucosal barrier-related proteins were determined to reveal the effects of fermented *D. candidum* polyphenols (FDC) on intestinal inflammation induced by oxazolone in zebrafish. The results showed that fermentation significantly changed the chemical composition of *D. candidum* and that FDC significantly improved the intestinal immune index. After the 21st day of FDC intervention, the abundance of *Lactobacillus, Faecalibacterium*, and *Rummeliibacillus* increased, but the abundance of the genera *Shewanella, Geodermatophilus, Peptostreptococcaceae*, and *Mycobacterium* decreased. At the same time, FDC significantly increased intestinal short-chain fatty acids (SCFAs). In addition, network analysis based on multi-omics indicated that FDC intake leads to changes in intestinal microbiota and intestinal metabolites, resulting in enhanced host immune function. These results indicate that FDC can improve intestinal health by regulating the intestinal microbiota and its metabolites to treat intestinal inflammation and regulate the host immune system. The present research improved our understanding of the utilization of *D. candidum* polyphenols and provided new evidence for the impacts of fermented *D. candidum* on host health.

## Introduction

Intestinal inflammation can occur in a variety of intestinal diseases, such as inflammatory bowel disease (IBD) (1), irritable bowel syndrome (IBS) (2), and intestinal ischaemia-reperfusion injury (3). Intestinal inflammation is a prominent feature of these diseases. The inflammatory response produces a large number of cytokines and inflammatory mediators, which reduce the activity of antioxidant enzymes, resulting in damage to tissues and intestinal epithelial cells; this ultimately causes intestinal barrier dysfunction and disrupts the balance of intestinal microbiota. Therefore, the treatment of intestinal inflammation may be an effective strategy for treating intestinal diseases. Increasing research has focused on the effect of the intestinal microbiota on intestinal diseases. Studies have proven that the intestinal microbiota can regulate immune factors, repair intestinal barrier function, and maintain intestinal environmental homeostasis (4, 5). SCFAs, an important metabolite of the intestinal microbiota, play an important role in immune regulation and intestinal barrier function. SCFAs have been shown to reduce gut inflammation by regulating T cells (6). SCFAs stimulate goblet cells to produce mucin, which in turn prevents pathogenic bacteria from adhering to intestinal epithelial cells (7). SCFAs also protect the intestinal barrier through coordinated regulation of tight junction proteins, which can maintain the integrity of intestinal epithelial cells, such as ZO-1 and occluding (8).

Polyphenols are found in fruits, vegetables, and grains, and they are a main natural compound; most polyphenols are considered to have potential biological activity in many diseases, such as cancer (9), obesity (10), diabetes, (11), and cardiovascular disease (12). Some active ingredients, such as polyphenols, change in most natural foods through fermentation, which is beneficial to promote the biological activity of foods, such as enhancing antioxidant activity and improving immune regulation and anti-inflammatory activity (13). Increasing studies have reported that polyphenols fermented by probiotics greatly change the composition of the intestinal microbiota (14). Polyphenols can regulate the intestinal microbiota by exerting prebiotic-like effects or antibacterial activity on pathogenic intestinal microbiota (15).

Zebrafish have become one of the most widely used animal models in biology because of their unique advantages. In recent years, there has been a significant increase in research on gastrointestinal diseases, especially in the study of the microbiota, the intestinal immune system, and other aspects. These studies found that the intestines of zebrafish are highly homologous to those of mammals in terms of development, tissue, and function (16–18). The intestinal epithelial cells of zebrafish and mammals are highly conserved in gene expression and transcriptional regulation (19), with many similar metabolic functions (20). The intestines of zebrafish and mammals are covered with a protective layer of mucin, the most important component of the intestine (21).

Zebrafish also have both innate and adaptive immune systems, which are very similar to the human immune system (22, 23). Macrophages, neutrophils, and eosinophils play a major role in the innate immune system in zebrafish and humans (24). Histocompatibility complex (MHC) class I or II molecules are also present in the adaptive immune system of zebrafish (25). The lymphatic system of zebrafish is similar to that of mammals in tissue, morphology and function, but it lacks some lymph nodes (26). Zebrafish is a good model for the study of intestinal diseases because the intestinal structure, molecular features and functions in zebrafish are similar to those in mammals. Studies have found that administration of oxazolone (4-ethoxymethylene-2-phenyl-2-oxazoline-5-one) in the intestinal tract of zebrafish can destroy the folded structure of the intestinal tract, deplete goblet cells, increase the infiltration of immune cells, and upregulate immune factors (27).

*Dendrobium candidum*, which is mainly distributed in tropical areas, belongs to the orchid family *Dendrobium*. *D. candidum* is a traditional Chinese medicinal material with high medicinal value and has been called “the first of the nine great immortal herbs.” *D. candidum* is a rare and endangered herb because of its strict environmental requirements, low natural reproduction capacity, and overexploitation. A thorough exploration of the functional activity of *D. candidum* and how to maximize its medicinal value have become primary objectives. Numerous studies have reported that *D. candidum* contains many active ingredients, including *Dendrobium* polysaccharides, alkaloids, polyphenols, phenanthrenes, anthraquinones, anthrones, and sesquiterpenoids (28–30).

At present, studies of *D. candidum* have mainly focused on its functional polysaccharide, which is related to antitumor, antiobesity and blood sugar-reducing activities (31, 32). However, the antioxidant, anti-diabetes, and anti-inflammatory effects of *Dendrobium* polyphenols have received limited attention (33). In addition, the large molecules of *D. candidum* can be decomposed into small molecules, which have greater biological activity because they are more conducive to human absorption, by fermentation, which also reduces economic costs. However, few studies have evaluated the *in vivo* activity and function of fermented *D. candidum* polyphenols, which inevitably interact with intestinal microorganisms, especially in the host. To address this challenge, the present research established an intestinal inflammation model in the model organism zebrafish. On the one hand, high-throughput metagenomic sequencing was applied to investigate the intestinal microbiota related to the utilization of *D. candidum* polyphenols. On the other hand, microbial metabolites, immune factors, apparent tissue morphology, and mucosal barrier-related proteins were analyzed to determine the effects of fermented *D. candidum* polyphenols on the intestinal microbiome-mediated immune response of zebrafish with intestinal inflammation. The present research extended our understanding of the utilization of *D. candidum* polyphenols by intestinal microbiota and provided new evidence for the impacts of fermented-*D. candidum* on host health.

## Materials and Methods

### The Fermentation and Determination of *D. candidum*

*D. candidum* fermentation broth was provided by the Department of Food Science and Engineering at Hainan University in China. The water extract of *D. candidum* was used as the culture medium, and *Lactobacillus plantarum* was used as the fermentation strain.

The fermentation broth of *D. candidum* was freeze-dried; 2 g of freeze-dried powder was weighed accurately and added to 20 mL of 80% methanol (Xilong, China). The solution was centrifuged at 5,000 rpm for 12 min, and the supernatant was collected. The supernatant was filtered through a 0.22 μm membrane and analyzed by high-performance liquid chromatography.

Polyphenol standard (34): Five polyphenol reference materials were used, including protocatechuic acid (PA), 4-hydroxybenzoic acid (HA), syringic acid (SA), p-hydroxycinnamic acid (PHA), and rutin (Solebo, China)

The conditions for liquid chromatography were as follows (34): chromatographic column, ACE Excel 3 C18-AR (3.0 μm, 4.6 x 150 mm); mobile phase, 10 mmol L^−1^ ammonium acetate solution (A); (Xilong, China) and methanol solution (B); detection wavelength, 260 nm; column temperature, 40°C; flow rate, 0.5 mL min^1^; injection volume, 10 μL; gradient elution conditions, see Supplementary Table 1.

### Animal Experiment

The 4-month-old adult zebrafish (*Danio rerio*), about 500 mg, used in this experiment were obtained from a local supplier in Haikou. The zebrafish were cultured at the aquatic experimental animal facility of the Department of Food Science and Technology of Hainan University. All animal experiments were performed in accordance with the “Code for the Care and Use of Experimental Animals of Hainan University” and approved by the Animal Ethics Committee of Hainan University. Before FDC exposure experiments, zebrafish were adapted to laboratory conditions in oxygenated and dechlorinated tap water for 14 days. According to the standard zebrafish feeding regimen, growth, and experiments were performed at 28 ± 0.5°C, with a light/dark cycle of 14/10 h, and commercial feed was fed at 3% of the total zebrafish daily weight (27, 35). After 14 days of acclimatization, all zebrafish were randomly divided into 80 glass water tanks (15 zebrafish/water tank), each with a capacity of 3 L of water, and randomly divided into 4 groups.

Some modifications were made to Sylvia Brugman's modeling method (27): fresh filtered water was supplemented with 10 mg L^−1^ oxazolone (oxa) (Far Field, China) in the first 3 days and 30 mg L^−1^ oxa in the next 3 days. The experiment was grouped as follows: (1) control group, fresh filtered water without FDC for 3 weeks; (2) FDC group, fresh filtered water supplemented with 0.15 g L^−1^ FDC for 3 weeks; (3) oxa group, fresh filtered water supplemented with 10 mg L^−1^ oxazolone in the first 3 days and 30 mg L^−1^ oxazolone in the next 3 days, then fresh filtered water without FDC for 3 weeks; (4) oxa + FDC group, fresh filtered water supplemented with 10 mg L^−1^ oxazolone in the first 3 days and 30 mg L^−1^ oxazolone in the next 3 days, then fresh filtered water supplemented with 0.15 g L^−1^ FDC for 3 weeks. During the entire exposure period, the water in each tank was replaced daily with fresh filtered water containing the corresponding concentration of FDC to ensure a constant FDC concentration in the water and daily removal of feces, inedible food, and dead fish.

### Collecting Samples

To avoid individual differences, three zebrafish intestines were mixed in one sample. Twenty-five samples were collected at each time point (0, 7, 14, and 21 days), totalling 75 zebrafish intestines. Five of the samples were immediately stored at −20°C for DNA extraction. Another 5 samples, totalling 15 intestines, were collected and immediately put into 4% paraformaldehyde overnight for intestinal tissue sections. The remaining 15 samples were weighed, frozen, homogenized, and centrifuged at 12,000 rpm for 20 min. The supernatant was collected and stored at −80°C for analysis of immune indicators.

### Intestinal Tissue Section

The method implemented for preparation of the intestinal section was slightly modified on the basis of experimental conditions, according to Qian (36). On days 0, 7, 14, and 21, the small intestine was obtained, immediately placed in 4% paraformaldehyde overnight, embedded in paraffin, and stained with haematoxylin and eosin (H&E). The fish intestines were treated as shown in Supplementary Tables 2, 3. Finally, the sections were observed under an optical microscope.

### Determination of Intestinal Barrier Indicators and Immune Indicators

The supernatant was tested for SIgA, MUC-2, occludin, ZO-1, TNF-α, IL-10, IL-4 (LMAI Biotechnology, China), and MPO (Enzyme-linked Biotechnology, China) by using the corresponding ELISA kit according to the manufacturer's instructions. The absorbance (OD value) was measured at a wavelength of 450 nm by using a microplate reader (SpectraMax M2, MD), and the content of the target indicator in the sample was calculated from the standard curve.

### Determination of Related Indexes of Intestinal Tissue Oxidative Stress

The supernatant was tested for the total proteins by using the total protein quantification kit (with standard, BCA method) according to the manufacturer's instructions. The levels of NO, SOD, GPX, and MDA (Nanjing Jianan, China) in tissues was determined with biochemical kits according to the manufacturer's instructions. The absorbance (OD value) was measured by using a microplate reader (SpectraMax M2, MD).

### DNA Extraction and Determination

The DNA of the zebrafish intestinal microbiota was extracted with a QIAGEN DNA Mini-Kit (Hilden, Germany). The quality of extracted DNA was determined by gel electrophoresis. Then, the DNA was quantified by an ultraviolet spectrophotometer. All of the isolated DNA was stored at −20°C and used as the template for further analysis.

Sequencing was conducted by Shanghai Personalbio Corporation. A set of 6-nucleotide barcodes was added to the universal forward primer 338F (5′-ACTCCTACGGGAGGCAGCA-3′) and the reverse primer 806R (5′-GGACTACHVGGGTWTCTAAT-3′), which target the V3–V4 variable region of the 16S rRNA gene. Referring to the preliminary quantitative results of electrophoresis, the PCR amplification product was subjected to fluorescence quantification, which was determined with the Quant-iT PicoGreen dsDNA Assay Kit, and the quantitative instrument was a Microplate reader (BioTek, FLx800). Next, the quantified amplified products were loaded onto the Illumina MISEQ high-throughput sequencing platform for sequencing (37).

### Analysis of Bacterial Composition

QIIME software (v1.9) was used to merge the obtained sequences and the sequences were clustered into OTUs according to 97% sequence similarity by using UCLUS (38). According to the abundance distribution of OTUs in different samples, the diversity level of each sample was evaluated. Each sample (group) at different classification levels was analyzed for specific composition (and whether there was a significant difference between groups).

### PICRUSt Functional Predictive Analysis

According to the full-length 16S rRNA gene sequence of the intestinal microbes, the gene function spectrum of their common ancestors was inferred. The gene function spectra of other unmeasured species in the Greengenes 16S rRNA gene full-length sequence database were inferred to construct a gene function prediction spectra of the entire lineage of archaea and bacteria. The 16S rRNA gene sequence data obtained by sequencing were compared with the Greengenes database, and the “nearest neighbor of the reference sequence” of each sequencing sequence was searched and classified as the reference OTU. According to the rRNA gene copy number of “the nearest neighbor of the reference sequence,” the obtained OTU abundance matrix was corrected. Finally, the microbiota composition data were “mapped” to the known gene function spectrum database to predict the metabolic function of the microbiota (39).

### Determination of SCFAs

According to a previous reference, 50 μL each of six SCFAs (including acetic acid, propionic acid, butyrate, isobutyric acid, valerate acid, and isovalerate acid) was added to a 10 mL volumetric flask with ether to a fixed volume (40). The concentration of the solution was 5 mL L^−1^. A total of 0.2~0.3 g of sample (accurate to 0.1 mg) was weighed and added to 2 mL of 0.5 mol L^−1^ sulfuric acid solution, which was with sealed. Then, the samples were extracted by ultrasonic vibration at 40°C at 35 kHz for 30 min. After cooling, 2 mL of diethyl ether (the level of liquid was recorded) was added to the samples, which were vigorously shaken for 5 min, and placed at 4°C for 30 min. Then, diethyl ether was added, and the solution was centrifuged at 12,000 rpm for 5 min. The upper ether solution was analyzed by GC-MS.

GC conditions were as follows: inlet temperature, 250°C; temperament interface temperature, 250°C; carrier gas flow rate, 1.5 mL min^−1^; split ratio, 3:1; injection volume, 1 μL; temperature program programme, initial 70°C, hold for 3 min, followed by an increase of 10°C min^−1^ to 100°C for 2 min, 8°C min^−1^ to 180°C for 0 min, then 10°C min^−1^ to 250°C for 15 min. MS conditions were as follows: ion source temperature, 230°C; quadrupole temperature, 150°C; EI ionization, 70 eV; full scan 35 ~ 550 da.

### Statistical Analysis

Related analysis of the output data was performed by R studio and GraphPad Prism software. The statistical analysis of SCFAs, biomarkers of oxidative stress, and immune or intestinal barrier indicators was conducted using Student's *t*-test. The analysis of beta diversity was performed using principal coordinates analysis (PCoA) based on UniFrac distance. A comparison analysis of PC1 was visualized with a boxplot (Wilcoxon rank-sum tests). Comparison analysis of the OTU differential relative abundance was conducted using the ANOVA test and Student's *t*-test, and the hierarchical cluster analysis (HCA) heat map was used for display. Comparison analysis of the metabolic pathways was conducted using Student's *t*-test. The Galaxy online analysis platform was used for LEfSe analysis of the metabolic functions of the intestine microbiota (41). The correlation network was established by Cytoscape to confirm the interactions among polyphenols, intestinal microbiota, metabolic pathways and immune indexes, and the Spearman rank correlation coefficient was estimated in R studio. The sequence data reported in this paper were deposited in the NCBI database (Metagenomic data: PRJNA593335).

## Results

### Effect of Fermentation on Polyphenols in *Dendrobium candidum*

The contents of several polyphenols were detected by high-performance liquid chromatography (HPLC) as shown in Table 1. The original HPLC peak was placed in the appendix of the article (Supplementary Figures 1, 2). The results showed that the contents of SA, HA, and PHA in FDC increased, while the contents of PA and rutin decreased, compared with the *D. candidum* extract (DC). Among them, HA increased by 13.3531 μg g^−1^, SA increased by 12.351 μg g^−1^, and PHA increased by 42.2914 μg g^−1^. These ingredients have antioxidant, anti-inflammatory, and anticancer effects, indicating that fermentation can improve the biological activity of FDC.

**Table 1:** Contents of five phenolic substances in FDC and DC.

**Ployphenol**	**FDC (μg g**^****−1****^**)**		**DC (μg g**^****−1****^**)**		***P*-value**
	**Average**	**SD**	**Average**	**SD**	
PA^**^	11.0535	1.4579	34.6081	5.0890	0.0048
HA^**^	26.2478	3.4629	12.8947	6.7367	0.0023
SA^**^	51.5758	6.2502	39.2248	15.3165	0.0090
Rutin^***^	2.1026	0.5417	51.7118	4.9121	<0.001
PHA^***^	58.1988	0.7986	15.9074	1.3168	<0.001

^*^p < 0.05;

** *p < 0.01*;

*** *p < 0.001*.

### FDC Improved Intestinal Physical Barrier Function

**Figure 1:**
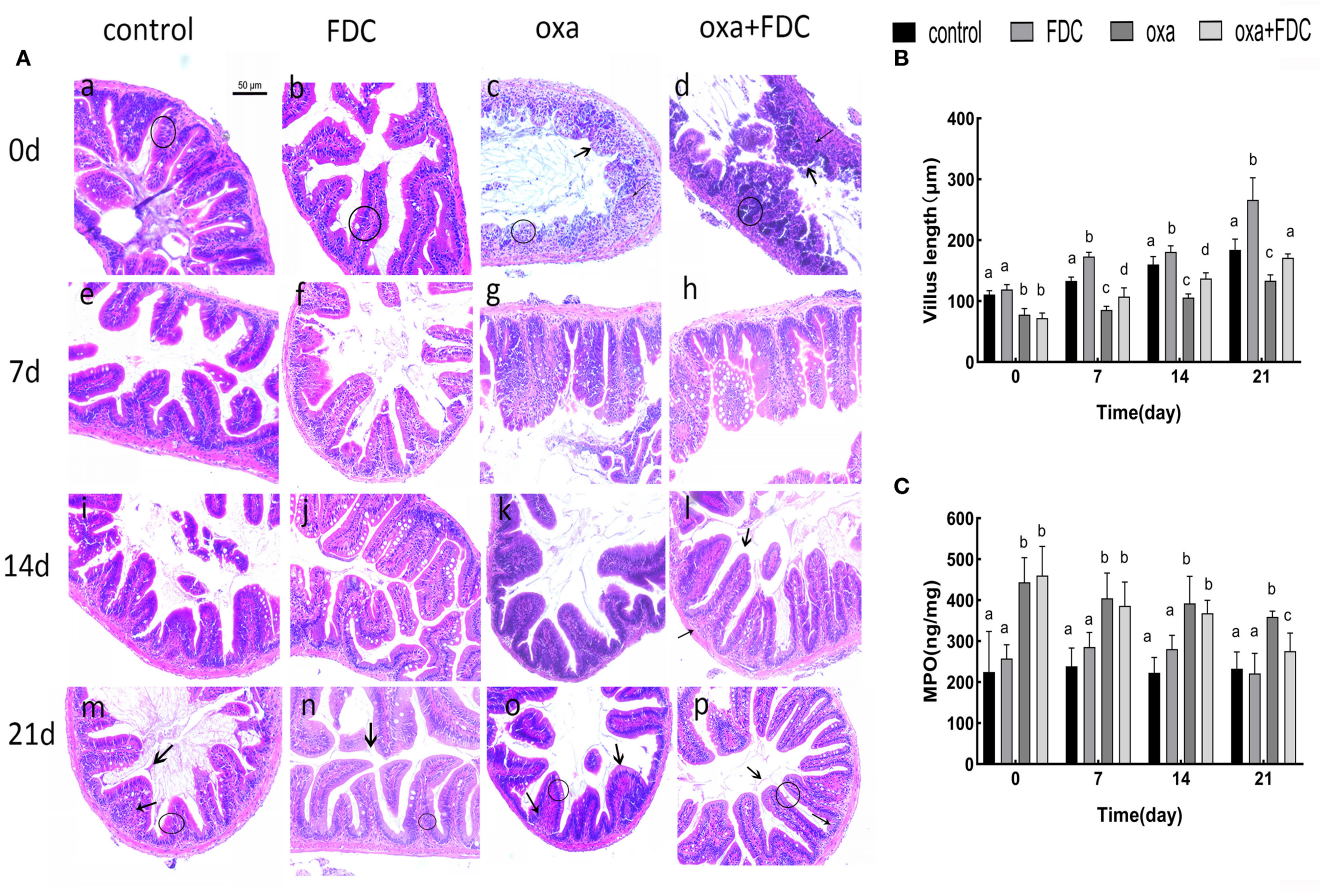
**(A)** Histological examination of intestines stained with H&E. Bars = 50 μm. “
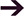
” indicates the intestinal villi, “
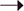
” indicates to inflammatory cells, and “◦” stands for columnar epithelial cells. **(B)** The mucosal thickness of intestinal segments. **(C)** Neutrophil symbol: MPO. Data are expressed as the mean ± SD. The same letter is not significantly different from each other (*P* < 0.05) on the same day.

H&E staining showed that after oral administration of FDC, the morphological structure of the intestinal mucosa in each group changed significantly (Figures 1A,B). The arrangement of columnar epithelial cells in the FDC group was more compact, and the height of villi increased significantly compared with the control group. The curative effect of FDC on intestinal inflammation was obvious compared with the oxa group.

On day 0, the infiltration of inflammatory cells was observed, and villi were seriously damaged in the oxa group. The infiltration of inflammatory cells in the oxa + FDC group decreased compared with that in the oxa group until day 14. Additionally, FDC treatment significantly enhanced the intestinal mucosal layer and significantly reduced the MPO content (*p* < 0.05) (Figure 1C).

**Figure 2:**
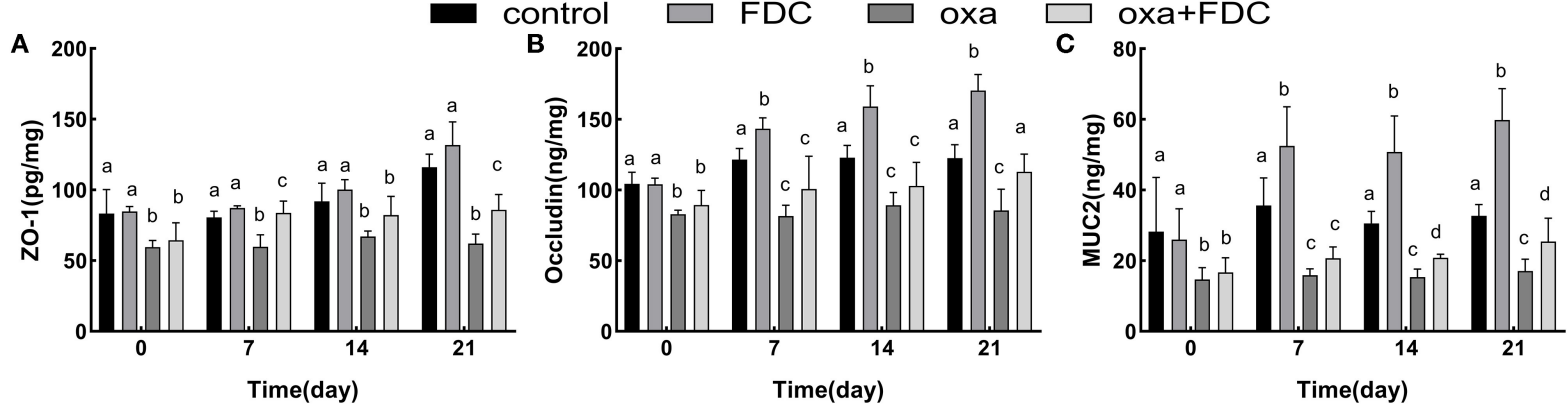
Comparative analysis of the typical intestinal mucosal barrier index **(A–C)**, including ZO-1, occludin and MUC2. Data are expressed as the mean ± SD. The same letter is not significantly different from each other (*P* < 0.05) on the same day.

The results of mucosal barrier indexes in intestinal tissues of different zebrafish groups after 21 days of experimental exposure are shown in Figure 2. After 6 days of oxa exposure, ZO-1, occludin and MUC2 were significantly lower in the oxa group than in the control group (*P* < 0.05). Occludin and ZO-1 are important proteins closely connected with intestinal tissue; these proteins increased 39 and 28% in the FDC group and 32 and 38.5% in the oxa + FDC group, respectively. MUC2 showed a significant upward trend during FDC exposure (*P* < 0.05); in the FDC group and oxa + FDC group, MUC2 increased 82 and 61%, respectively. These data suggest that FDC exposure affects the intestinal barrier function of the host. FDC exposure promoted the secretion of MUC2, which implied that it is beneficial to isolate the intestinal epithelial tissue from contact with pathogenic bacteria compared with the control group. The increase in occludin and ZO-1 indicates that FDC facilitates tight junctions between intestinal epithelial cells to maintain integrity of the intestinal physical barrier. Oxa exposure resulted in disturbances in the secretion of occludin and ZO-1, increasing the permeability of the intestinal epithelium and leading to the destruction of the intestinal barrier. A lack of MUC2 can lead to the direct contact of pathogens with intestinal epithelial cells, resulting in intestinal inflammation. After 21 days of FDC treatment, the contents of occludin, ZO-1 and MUC2 increased, indicating that FDC effectively prevented pathogens from invading intestinal epithelial cells and repaired the intestinal barrier.

### FDC Stimulated Intestinal Immune Responses

**Figure 3:**
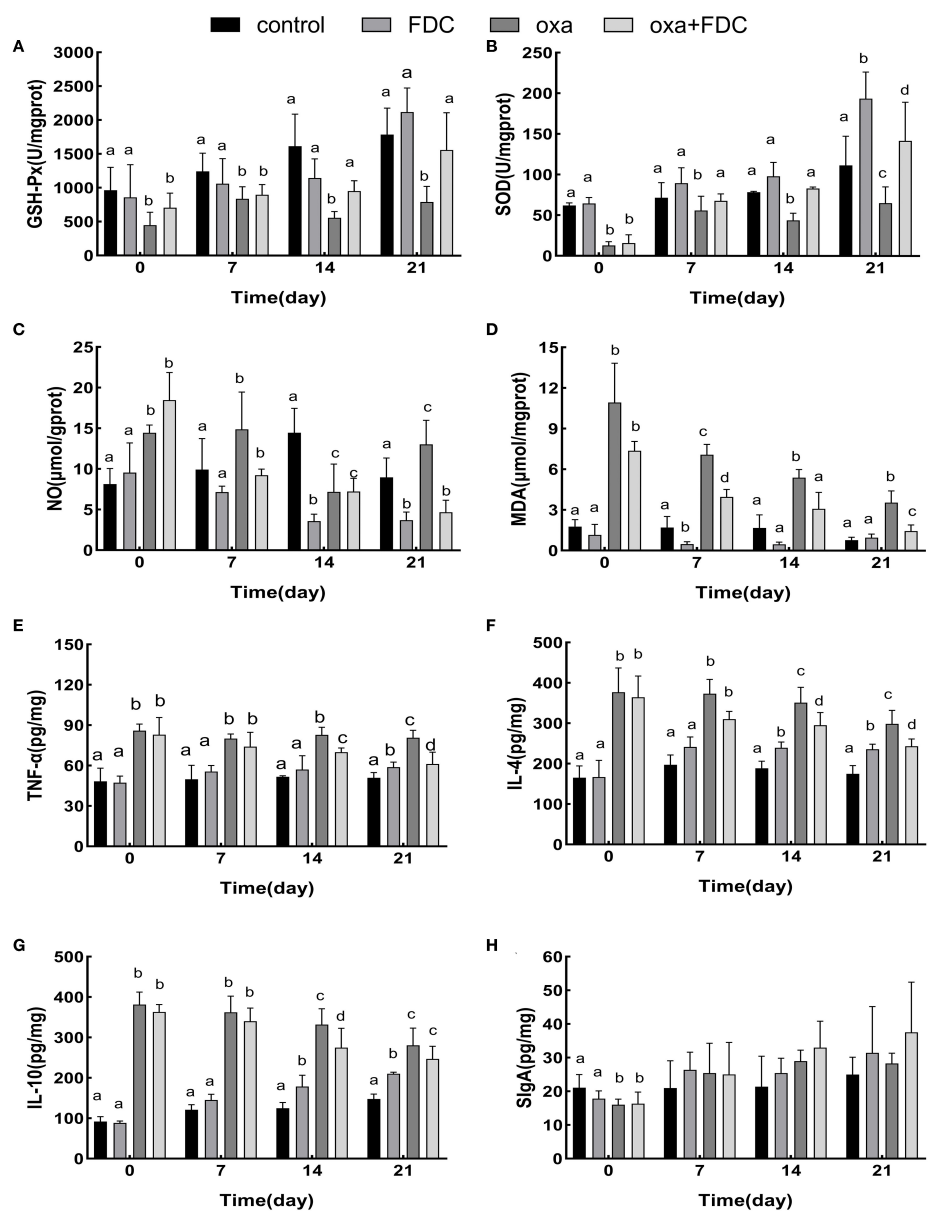
Comparative analysis of the intestinal immunity index **(A–H)**. Data are expressed as the mean ± SD. The same letter is not significantly different from each other (*P* < 0.05) on the same day.

Malondialdehyde (MDA), nitric oxide (NO), superoxide dismutase (SOD), and glutathione peroxidase (GSH-Px), as markers of oxidative stress, showed significant changes 21 days after FDC exposure (Figures 3A–D). The results showed that after oxazolone exposure, the contents of MDA and NO increased significantly, and the activities of SOD and GSH-Px decreased at day 0. MDA and NO decreased in the FDC group (34 and 58%, respectively), and oxa + FDC group (79 and 72%, respectively). After 14 days, the activity of SOD (82.93 ± 2.05 U/mg prot) (*P* < 0.001) and the activity of GSH-Px (954.07 ± 153.26 U/mg prot) (*P* < 0.01) in the oxa + FDC group increased significantly. FDC exposure increased the content of SOD and GSH-Px, and decreased the content of NO and MDA, implying that FDC enhanced the antioxidant defense mechanism of intestinal cells and delayed the cell damage caused by intestinal inflammation.

At the same time, TNF-α, IL-4, and IL-10 cytokines significantly changed as a result of FDC exposure (Figures 3E–H). Compared with the control group, the levels of IL-10 and IL-4 in the colon tissue of the FDC group increased 1.3 times and 41%, respectively. The cytokine secretion of the oxa group was significantly lower than that of the control group at day 0. In the case of inflammation, FDC treatment significantly reduced the levels of IL-4 by 35% and levels of TNF-α by 27%. The SIgA of intestinal immunoglobulin increased during FDC exposure, but there was no significant difference. Compared with the control group, the increase in IL-10, IL-4, and TNF-α in the FDC group indicates that FDC can be directly stimulate the host's immune response and improve immune capacity. After the model of intestinal inflammation was induced, the addition of FDC reduced the secretion of IL-10, IL-4, and TNF-α, indicating that FDC can slightly relieve intestinal inflammation.

### The Intestinal Microbiome and Its Metabolites Responded to FDC Intervention

**Figure 4:**
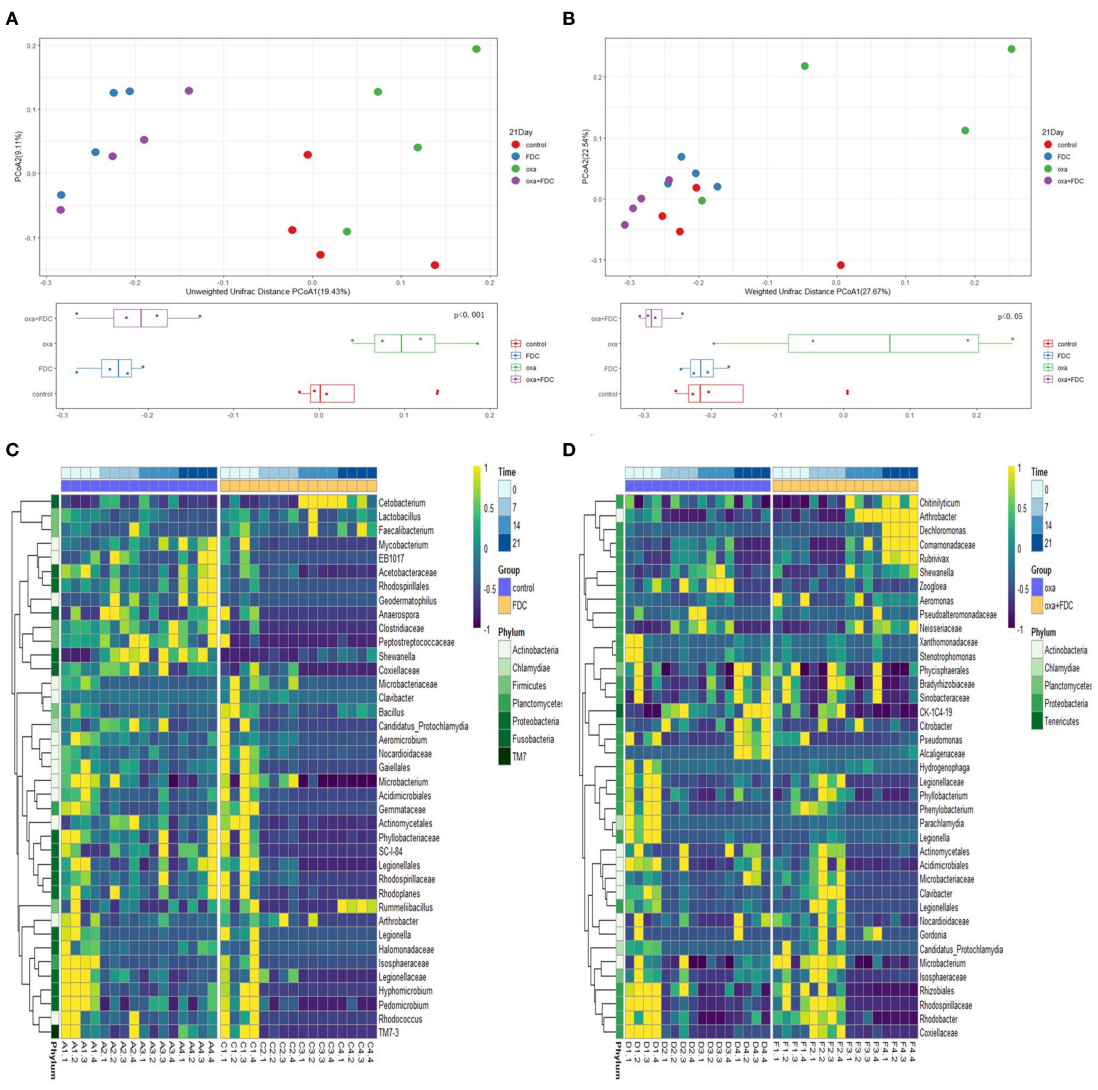
Effects of FDC administration on the gut microbiota composition. **(A,B)** A principal component (PCoA) score plot based on unweighted and weighted UniFrac metrics for all samples on day 21. Each point represents the composition of the intestinal microbiota of one sample. **(C,D)** Heatmap constructed using the number of significantly different species on days 0, 7, 14, and 21.

The high-throughput sequencing method was used to research the classification and functional characteristics of the intestinal microbiota, as shown in the figure. According to the OTU data of the sequencing run, on days 0, 7, 14, and 21, the trajectories of the four groups of intestinal microbiota were determined by weighted and unweighted UniFrac PCoA (Figures 4A,B and Supplementary Figure 3). UniFrac PCoA showed that after 21 days of treatment, the gut microbiota structure of the FDC group had already significantly diverged from that of the control group. Meanwhile, the bacterial structure of the oxa + FDC group was highly similar to that of the FDC group.

The results indicated that FDC had a significant effect on intestinal microbiota. As shown in the figures (Supplementary Figure 4 and Figures 4C,D), after 21 days, *Lactobacillus, Faecalibacterium*, and *Rummeliibacillus* were enriched in the FDC group. The abundance of *Peptostreptococcaceae* and *Mycobacterium* decreased significantly. At the same time, FDC significantly reduced the abundance of *Citrobacter, Pseudomonas*, and *Alcaligenaceae*. At the functional level, we carried out predictive functional analysis on the 16S rDNA sequence and functional path analysis at four time points (including the third level defined by KEGG) by using LEfSe.

**Figure 5:**
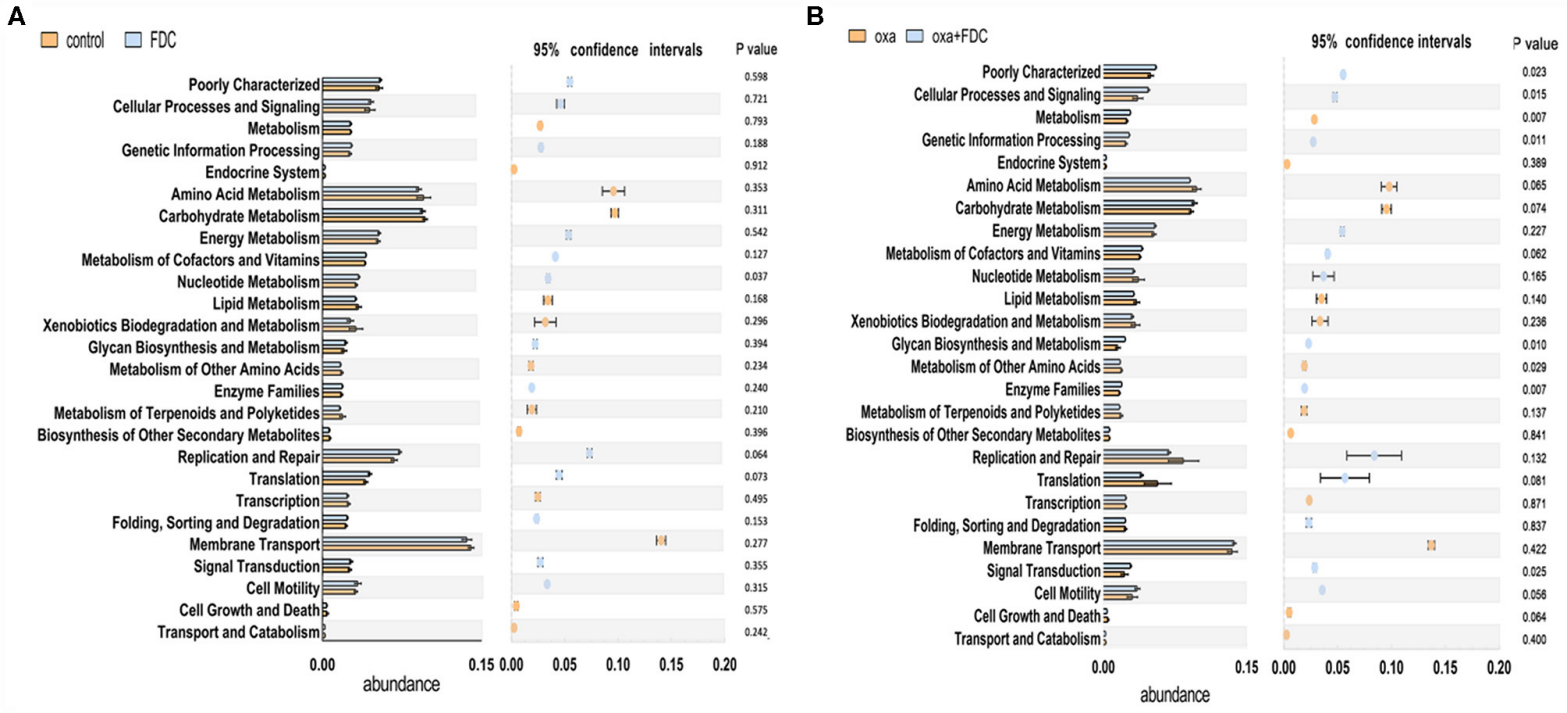
Analysis of the relative abundance of metabolic pathways encoded in each input sample metagenome on day 21. **(A)** The control group and the FDC group were compared with the KEGG second-level functional group, **(B)** The oxa group and the oxa+FDC group were compared with the KEGG second-level functional group.

**Figure 6:**
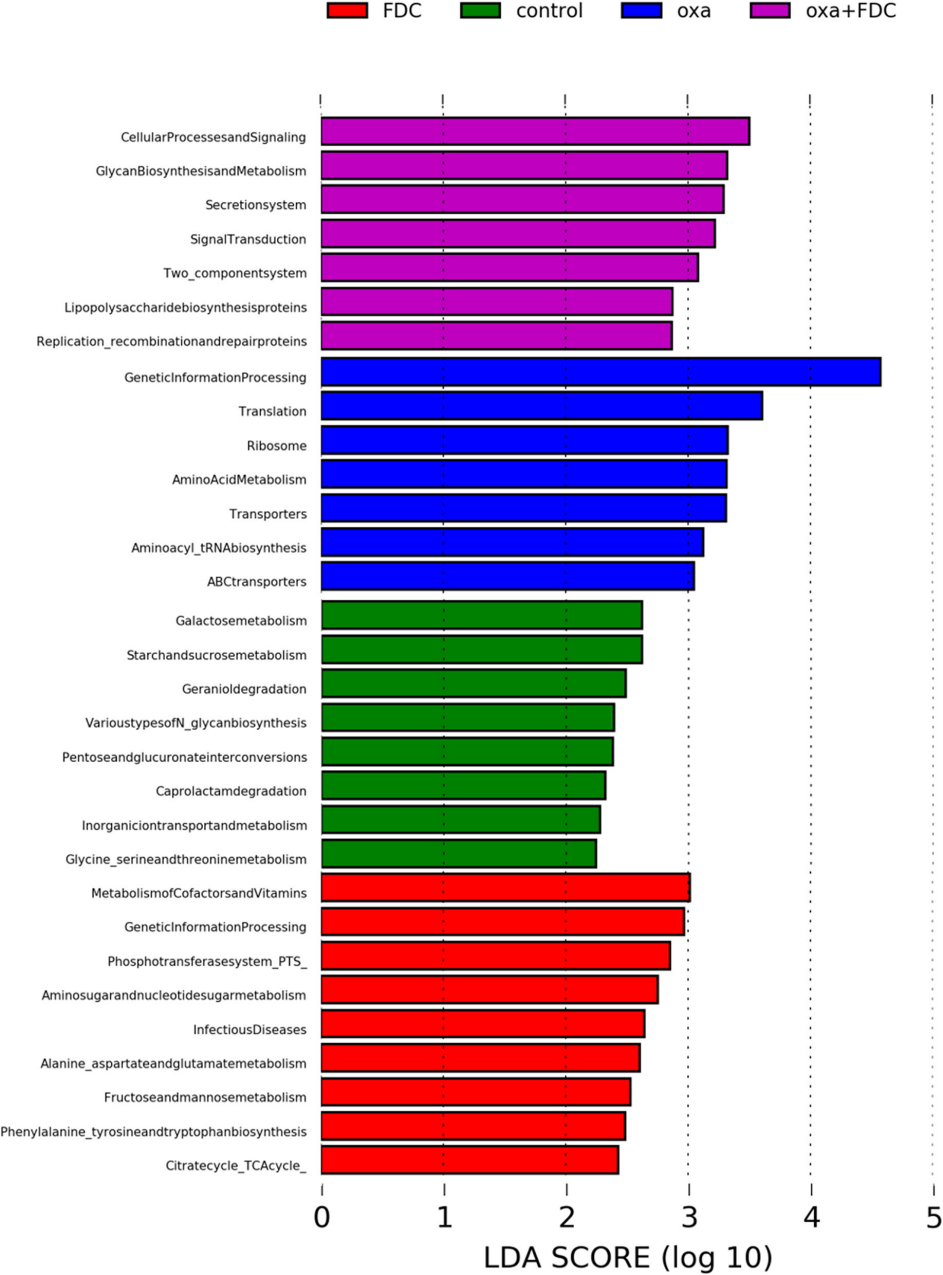
Analysis of differences in KEGG metabolic pathways on day 21.

The KEGG pathway analysis showed that membrane transport, replication and repair, energy metabolism, carbohydrate metabolism, and amino acid metabolism accounted for the highest proportion of the top five pathways. At the same time, the pathways with the largest significant difference between the oxa group and the oxa + FDC group were metabolism, metabolism of other amino acids, genetic information processing, and glycan biosynthesis and metabolism (Figure 5). After 21 days, the differentially expressed genes in the FDC group were involved in the phospho transferase system (PTS), citrate cycle (TCA cycle), genetic information processing, cellular processes and signaling, two component system and replication recombination and repair proteins, and these differentially genes are abundant in many KEGG pathways (Figure 6). In addition, the differentially expressed genes between the control group and the FDC group and the oxa group and the oxa + FDC group at different times according to KEGG pathway analysis are shown in Supplementary Figures 5, 6. As shown in the figures, the differentially expressed genes were mainly concentrated in amino acid-related metabolism, signaling molecules, and interactions. After 14 days, the differentially expressed genes were mainly enriched in the genetic information processing-related pathway. Although the abundance of genes may have been different at different time points, the number of differentially expressed genes increased significantly over time (Figures 5, 6 and Supplementary Figures 5, 6).

### Effects of FDC on SCFAs

**Figure 7:**
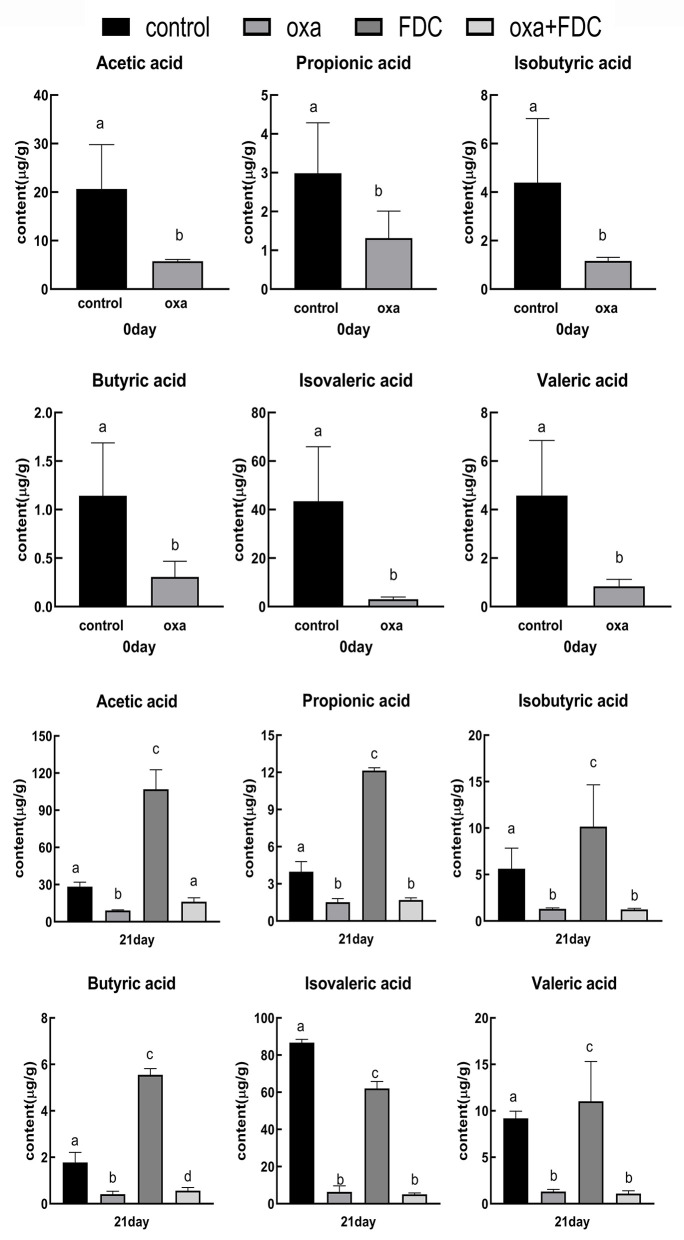
Differences in short chain fatty acids between groups. Data are expressed as the mean ± SD. The same letter is not significantly different from each other (*P* < 0.05) on the same day.

Six major SCFAs, acetic acid, propionic acid, isobutyric acid, butyric acid, isovaleric acid, and valeric acid, were selected and measured in the intestinal samples. The acetic acid, propionic acid, and butyric acid concentrations were remarkably decreased by oxa administration after 0 days (control vs. oxa, *P* < 0.05, Figure 7). In comparison, FDC administration noticeably increased the levels of acetic acid, propionic acid, isobutyric acid, and butyric acid after 21 days (oxa vs. oxa + FDC, *P* < 0.05; control vs. FDC, *P* < 0.05, Figure 7). However, no significant differences were observed in the production of propionic acid, isobutyric acid, isovaleric acid, and valeric acid (oxa vs. oxa + FDC, *P* > 0.05; Figure 7).

### FDC Relieved Intestinal Inflammation Through the Intestinal Microbiome Mediated Immune Response

**Figure 8:**
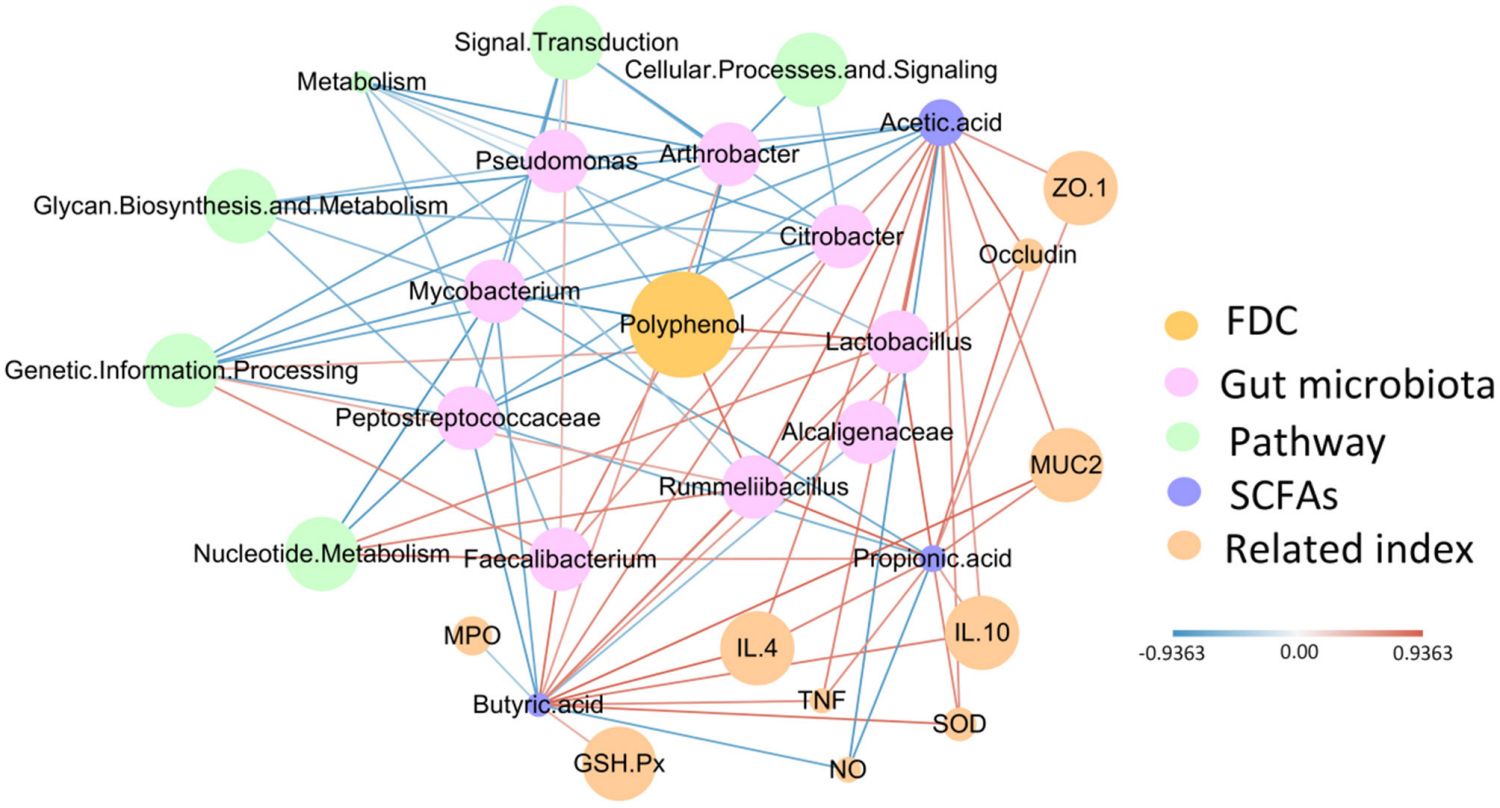
The correlation network constructed with polyphenols, bacterial species, microbial metabolic pathways, SCFAs, the immune index, biomarkers of oxidative stress and the physical barrier index. The edge width and color (red, positive and blue, negative) are proportional to the correlation strength. The node size is proportional to the relative mean abundance.

Based on the above phenomenon, we found that FDC improved intestinal barrier function, stimulated the immune response, and changed the intestinal microbiota. At the same time, we also found that FDC relieved intestinal inflammation. Therefore, to more clearly define the role of FDC in improving the immune response to intestinal inflammation, we constructed a radial network diagram with FDC as the core (Figure 8). From the figure, we observed that FDC exposure can promote the growth of *Lactobacillus, Faecalibacterium*, and *Rummeliibacillus*, which were positively correlated with SCFAs and had a strong positive correlation with nucleotide metabolism and genetic information processing. SCFAs were positively correlated with indicators of inflammation and physical barriers. At the same time, FDC exposure inhibited the growth of *Citrobacter, Pseudomonas, Alcaligenaceae, Peptostreptococcaceae*, and *Mycobacterium*. These bacteria had a strong negative correlation with SCFAs. Therefore, we conclude that FDC exerts beneficial effects through the host intestinal microbiota and its metabolites.

## Discussion

In the present study, a model of intestinal inflammation was established with the help of the model organism zebrafish, and the effects of FDC on the composition of the intestinal microbiota and the immune regulation of the intestine were evaluated.

The polyphenols in *D. candidum* significantly changed during fermentation, suggesting that the changes in intestinal microbiota composition and inflammation regulation might be due to the effect of polyphenols. Polyphenols are a well-known class of active substances, and their antioxidant activity and anti-inflammatory activity have long been confirmed. After fermentation by *Lactobacillus plantarum*, polyphenols are further decomposed into small molecular active substances, which may improve the bioavailability and bioactivity of *D. candidum*. HPLC analysis showed that after fermentation of *D. candidum*, the composition of polyphenols changed greatly, and the content of SA, HA, and PHA increased significantly. The beneficial effects of PHA in improving oxidative stress conditions, especially in the intestinal inflammatory response, were observed (42). SA has antioxidant, anti-inflammatory, antibacterial, and other effects and has a wide range of therapeutic effects in the prevention of diabetes, cancer, and cardiovascular disease (43).

Oxazolone can cause Th2-type inflammation mediated by IL-4, which can lead to the aggregation of effector T cells and the release of a large number of cytokines, such as IL-10 and TNF-α (44, 45). Severe inflammation can lead to damage to intestinal cells, exacerbating the oxidative stress response and ultimately damaging the intestinal barrier. In our study, after oxa administration, IL-4, IL-10, and TNF-α were enriched, which was consistent with previous reports. After treatment with FDC, the levels of IL-4, IL-10, and TNF-α were decreased. Oxazolone caused intestinal mucosal injury, decreased the levels of goblet cells and their mucin, reduced the contents of occludin and ZO-1, and increased the permeability of intestinal epithelial cells. After FDC exposure, intestinal epithelial cells were closely arranged, the number of goblet cells increased, and the levels of MUC2, occludin, and ZO-1 increased. As reported in previous studies, neutrophil infiltration was observed in rectal biopsies from patients with UC, and DSS-induced colitis was characterized by a significant increase in neutrophil accumulation in the colon (46, 47). Similarly, in our experiment, oxa caused neutrophil aggregation and increased MPO activity, while FDC significantly inhibited neutrophil enrichment.

Intestinal cells have an effective antioxidant defense mechanism that can resist oxidative damage. SOD and GSH-Px are the main substances of the antioxidant mechanism that can remove free radicals, resist the damage of free radicals, and repair damaged cells. Damaged intestinal cells caused by intestinal inflammation lead to decreased activity of GSH-Px and SOD, inducing lipid peroxidation and producing lipid peroxidation products such as MDA. The content of MDA and the activity of GSH-Px and SOD can quantitatively reflect the degree of intestinal inflammation. After FDC treatment, the activity of SOD and GSH-Px increased significantly, and the content of MDA and NO decreased significantly. This suggests that FDC may protect against oxidative damage by upregulating the activity of SOD and GSH- Px, enhance the antioxidant defense mechanism of intestinal cells, and delay the cell damage caused by intestinal inflammation.

High-throughput sequencing results showed that after the administration of oxa, the structure of the intestinal microbiota significantly changed. There was no significant change in alpha diversity of intestinal microbiota after the administration of FDC (Supplementary Table 4). FDC exposure had a significant effect on the intestinal microbiota. After 21 days, compared with the control group and the oxa group, there were significant differences in the intestinal microbiota and metabolic pathways of the FDC group and the oxa + FDC group, respectively. The main microbe-level change caused by FDC exposure was the decrease in bacterial content, and a small number of bacteria showed an upward trend. *Lactobacillus* and *Faecalibacterium* (48), as beneficial gut bacteria, produce SCFAs and other metabolites through the fermentation of polyphenols, inhibiting the growth of pathogenic bacteria and playing a beneficial role in regulating the intestinal tract and the host's immune system. *Faecalibacterium* is the most important and abundant bacteria that produces butyrate in the intestine. Consistent with these findings, *Faecalibacterium* was significantly enriched in zebrafish treated with FDC in our study. Moreover, butyric acid production was markedly increased after FDC administration and positively correlated with an increase in *Faecalibacterium* abundance. *Rummeliibacillus stabekisii* improved the growth, immunity, and disease resistance of fishes (49). In our study, FDC increased the abundance of *Lactobacillus, Faecalibacterium*, and *Rummeliibacillus*, which was beneficial to the stability of the intestinal health environment.

In this study, combined with the functional analysis of the microbiota, we found that the related pathways of genetic information processing and cellular processes were two of the top pathways at each time point. This finding indicated that FDC treatment interfered with the cell composition and growth process and upregulated the expression of related genetic genes; this observation is worthy of further study. We focused on the TCA cycle and SCFA metabolism. Some studies have found that in the case of intestinal inflammation, the intestinal microbiota is maladjusted, leading to a decrease in the metabolic products required in the TCA cycle. The downregulation of the TCA cycle reduces the amount of energy for intestinal epithelial cells (50). In our study, after 21 days of FDC exposure, the TCA cycle was significantly downregulated, suggesting that the intestinal microbiota decomposes FDC and provides energy for the life activities of intestinal cells through the TCA cycle.

SCFAs are the main metabolite of the intestinal microbiota and the main energy source of cells. Propionate can also promote dendritic cells to induce FOXP3 expression in T cells (51). Butyrate induces the binding of GPR109A, a G protein-coupled receptor, to mediate the action of colonic dendritic cells and macrophages, thus inducing the secretion of IL-10 and IL-4 and then activating T-reg cells, which play an immunomodulatory role; this result has been confirmed in another study (52). SCFAs were positively correlated with IL-4 and IL-10 and negatively correlated with TNF-α, suggesting that FDC, after fermentation by *Faecalibacterium*, produced SCFAs, regulated immune factors, and reduced the level of inflammation, which was also observed in previous studies.

In addition, *Faecalibacterium* are associated with intestinal mucus levels and intestinal barriers due to their production of SCFAs (53). This is mainly reflected in the regulation of mucin secretion and the tight junction of intestinal epithelial cells (7, 8). These results that FDC may regulate the intestinal barrier and repair the damaged mucosa by producing SCFAs through *Faecalibacterium*.

Meanwhile, in this study, we found that after FDC exposure, *Peptostreptococcaceae* started to decline after 7 days. *Peptostreptococcaceae* is enriched in the intestinal tract of patients with colon cancer, and polyphenol exposure reduces *Peptostreptococcaceae* in a dose-dependent manner (54). *Mycobacterium* and *Clostridiaceae*, as pathogenic bacteria, showed a downward trend after 7 days of exposure to FDC, which is consistent with the conclusion of Anand et al. (55). Meanwhile, in the oxa + FDC group, FDC administration inhibited the growth of *Citrobacter* and *Pseudomonas* after 21 days. *Citrobacter* and *Pseudomonas* are both intestinal pathogens that can induce intestinal inflammation under certain conditions. Polyphenols reduced the abundance and pathogenicity of *Citrobacter, Pseudomonas*, and *Clostridium* (56–58). Due to the different cell membrane structures of gram-positive bacteria and gram-negative bacteria, the mechanism of action of dietary polyphenols is different. Polyphenols can bind to the cell membrane of bacteria in a concentration-dependent manner or enhance/inhibit enzyme activity to change the structure and function of the cell membrane and then inhibit the growth of bacteria (59, 60).

The purpose of this study was to evaluate the effect of FDC on the intestinal microbiota of zebrafish, and to explore the mechanism of *D. candidum* polyphenols in alleviating zebrafish intestinal inflammation through intestinal microbiota-mediated immune responses. The balance between immune tolerance and inflammation is partially regulated by the interaction between innate and adaptive immune cells and intestinal microbiota. Under normal circumstances, FDC can generate metabolites such as SCFAs through the fermentation and decomposition of beneficial intestinal bacteria, stimulate the secretion of cytokines and activate the immunoregulation of immune cells. At the same time, in the case of intestinal inflammation, FDC can restore the balance of intestinal microbiota and regulate the secretion of immune factors, oxidative stress markers and intestinal barrier indicators. Therefore, the immune system was restored to normal levels, and the damaged intestinal mucosal barrier was repaired. By considering the effect of FDC on the dynamic balance of intestinal microorganisms and the immune system, this study provides a new perspective for evaluating the biological activity of FDC, contributing to further understanding the impact of FDC on intestinal function. Simultaneously, our study provides a theoretical basis for the study of the impact of FDC on human health. However, although we have demonstrated the beneficial effect of FDC on the intestinal flora and immune system in zebrafish, FDC also has a significant delaying effect on intestinal inflammation. Meanwhile, we have confirmed the effectiveness of the zebrafish model. However, at the same time, we considered the applicability of the results of this study in mammals such as mice. Therefore, we will consider extending the present research results to mouse models in our next study.

## Data Availability Statement

The datasets presented in this study can be found in online repositories. The names of the repository/repositories and accession number(s) can be found below: https://www.ncbi.nlm.nih.gov/, PRJNA593335.

## Ethics Statement

The animal study was reviewed and approved by the Ethics Committee of Hainan University.

## Author Contributions

DX and JZ conceived and designed the experiments. XG, SJ, and HT performed the experiments and analyzed the data. XG and JZ wrote and revised the manuscript. All authors read and approved the final manuscript.

## Conflict of Interest

The authors declare that the research was conducted in the absence of any commercial or financial relationships that could be construed as a potential conflict of interest.

## Supplementary Material

The mobile phase gradient program of HPLC is showed in Supplementary Table 1. Dying pretreatment and dyeing treatment is showed in Supplementary Tables 2, 3. The effect of FDC on the biodiversity of the oxa-induced zebrafish intestinal inflammation model is showed in Supplementary Table 4. The original HPLC peak is placed in the appendix of the article, as shown in Supplementary Figures 1, 2. A principal component (PCoA) score plot based on unweighted and weighted UniFrac metrics for all samples at different time points, as shown in Supplementary Figure 3. Structural changes of intestinal flora on the phylum in each group within 21 days is shown in Supplementary Figure 4. KEGG analysis of genes differentially expressed in each group of tertiary metabolic pathways at different time points, as shown in Supplementary Figures 5, 6.
