# Supplementary material for: Polyphenols in the Fermentation Liquid of Dendrobium candidum Relieve Intestinal Inflammation in Zebrafish Through the Intestinal Microbiome-Mediated Immune Response
Source: Front Immunol. 2020 Jul 17;11:1542. doi: 10.3389/fimmu.2020.01542 (PMC7379839; doi:10.3389/fimmu.2020.01542)
Supplement: Supplementary file 1 [file Data_Sheet_1.docx]

Supplementary Material

# Supplementary Data

## Supplementary Figures

**Table 1.** The mobile phase gradient program of HPLC

| Time/min | 10mmol/L Ammonium acetate/% | methanol/% |
| --- | --- | --- |
| 0.0 | 95 | 5 |
| 2.0 | 88 | 12 |
| 7.0 | 88 | 12 |
| 12.0 | 55 | 45 |
| 20.0 | 20 | 80 |
| 25.1 | 20 | 80 |
| 26.0 | 95 | 5 |
| 30.0 | 95 | 5 |

**Table 2.** Dyeing treatment

| Reagent | Time |
| --- | --- |
| xylene Ⅰ solution | 10min |
| xylene Ⅱ solution | 10min |
| 1: 1 alcohol-xylene solution | 15min |
| the ethanol of 100% | 5min |
| 90% | 5min |
| 80% | 5min |
| 70% | 5min |
| 50% | 5min |
| water | 5min |
| hematoxylin | 5 min |
| 1% hydrochloric acid alcohol | 5 s |
| water | 2 min |
| 70% alcohol | 2 min |
| 80% alcohol | 2 min |
| 90% alcohol | 2 min |
| 95% alcohol | 2 min |
| 0.5% eosin staining solution | 5 min |
| 95% alcohol | 2 min |
| 100% alcohol Ⅰ | 2 min |
| 100% alcohol Ⅱ | 2 min |
| 1: 1 alcohol-xylene | 2 min |
| xylene Ⅰ | 2 min |
| xylene Ⅱ | 2 min |

**Table 3.** The effect of FDC on the biodiversity of the oxa-induced zebrafish enteritis model.

|  |  | Simpson |  | Chao1 |  | ACE |  | Shannon |  |
| --- | --- | --- | --- | --- | --- | --- | --- | --- | --- |
|  | Parameter | average | SD | average | SD | average | SD | average | SD |
| Group | Time（day) |  |  |  |  |  |  |  |  |
| control | 0 | 0.86 | 0.05 | 785.67 | 118.00 | 818.49 | 124.04 | 5.38 | 0.48 |
|  | 7 | 0.85 | 0.03 | 875.90 | 80.51 | 938.79 | 103.84 | 4.90 | 0.39 |
|  | 14 | 0.87 | 0.04 | 806.53 | 73.81 | 905.86 | 82.01 | 4.93 | 0.59 |
|  | 21 | 0.82 | 0.05 | 964.10 | 192.14 | 1017.62 | 220.83 | 5.10 | 0.35 |
| oxa | 0 | 0.95 | 0.02 | 848.47 | 148.00 | 884.22 | 164.26 | 6.15 | 0.28 |
|  | 7 | 0.85 | 0.03 | 875.90 | 80.51 | 938.79 | 103.84 | 4.90 | 0.39 |
|  | 14 | 0.86 | 0.03 | 863.89 | 63.91 | 911.76 | 62.80 | 4.84 | 0.35 |
|  | 21 | 0.81 | 0.06 | 655.33 | 119.90 | 695.68 | 135.02 | 4.05 | 0.38 |
| FDC | 7 | 0.80 | 0.08 | 714.00 | 57.96 | 730.17 | 41.34 | 4.41 | 0.49 |
|  | 14 | 0.89 | 0.02 | 797.00 | 47.91 | 849.17 | 44.59 | 4.92 | 0.38 |
|  | 21 | 0.86 | 0.04 | 873.03 | 119.45 | 912.08 | 111.83 | 4.93 | 0.17 |
| oxa+FDC | 7 | 0.80 | 0.08 | 714.00 | 41.34 | 730.17 | 41.34 | 4.41 | 0.49 |
|  | 14 | 0.84 | 0.02 | 796.62 | 86.51 | 845.08 | 95.56 | 4.66 | 0.08 |
|  | 21 | 0.70 | 0.07 | 747.35 | 59.33 | 785.11 | 60.83 | 3.98 | 0.36 |

## Supplementary Figures


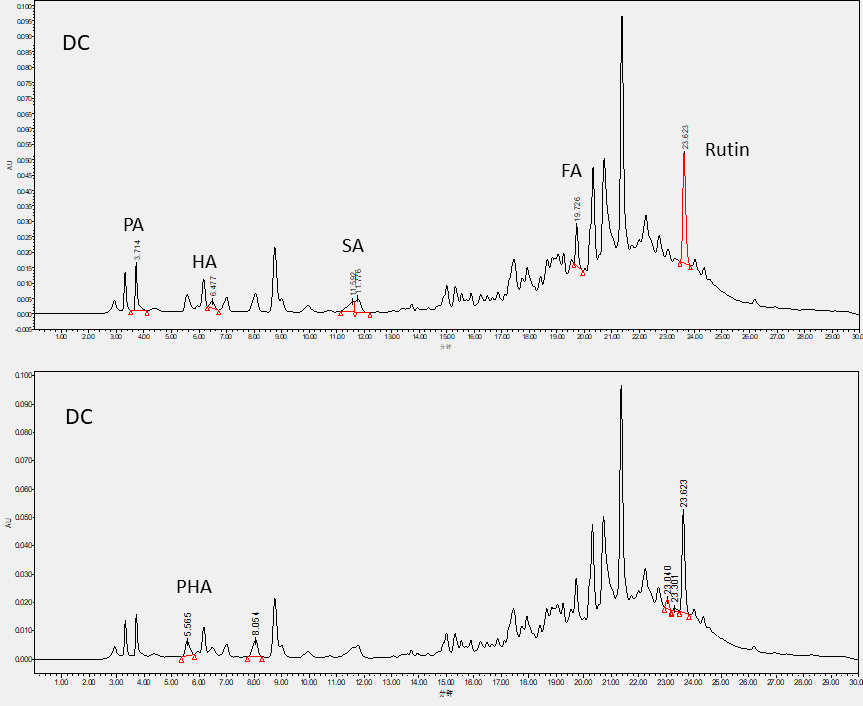


**Supplementary Figure 1.** High Performance Liquid Chromatogram of DC


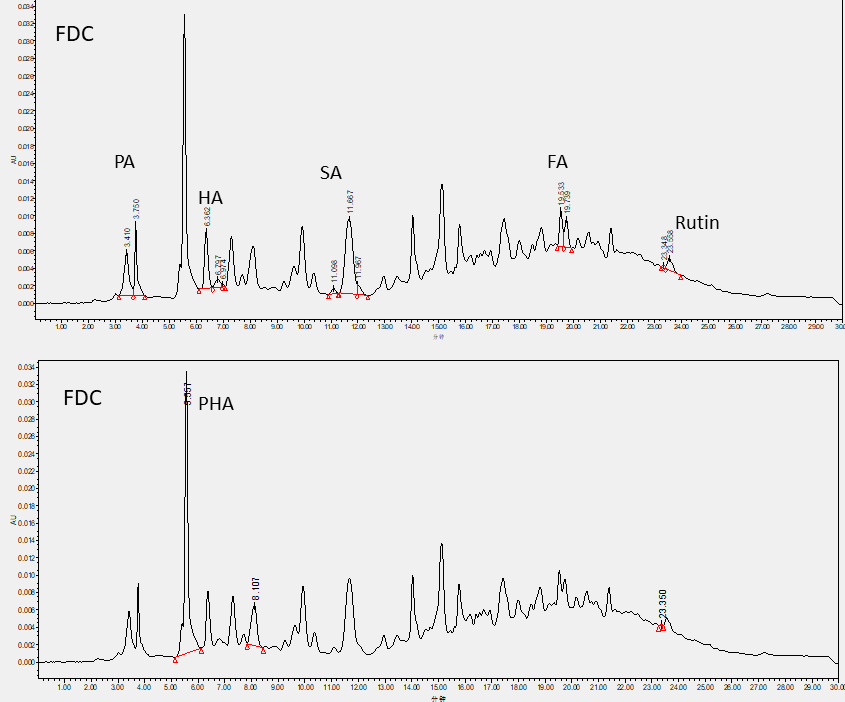


**Supplementary Figure 2.** High Performance Liquid Chromatogram of FDC


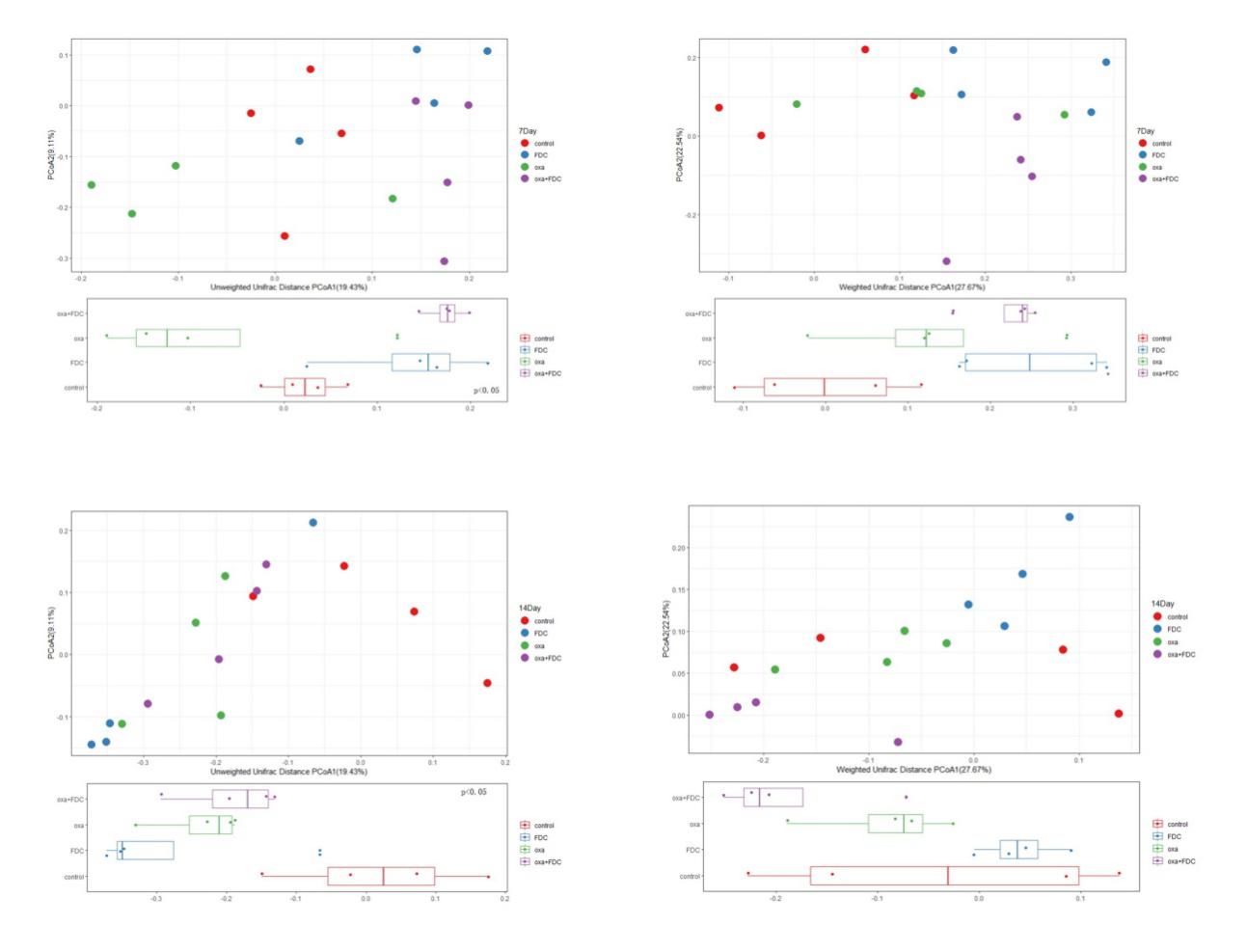


**Supplementary Figure 3.** Effects of FDC administration on the gut microbiota composition. A principal component (PCoA) score plot based on unweighted and weighted UniFrac metrics for all samples. Each point represents the composition of the intestinal microbiota of one sample.

**Supplementary Figure 4.** Structural changes of intestinal flora on the phylum in each group within 21 days


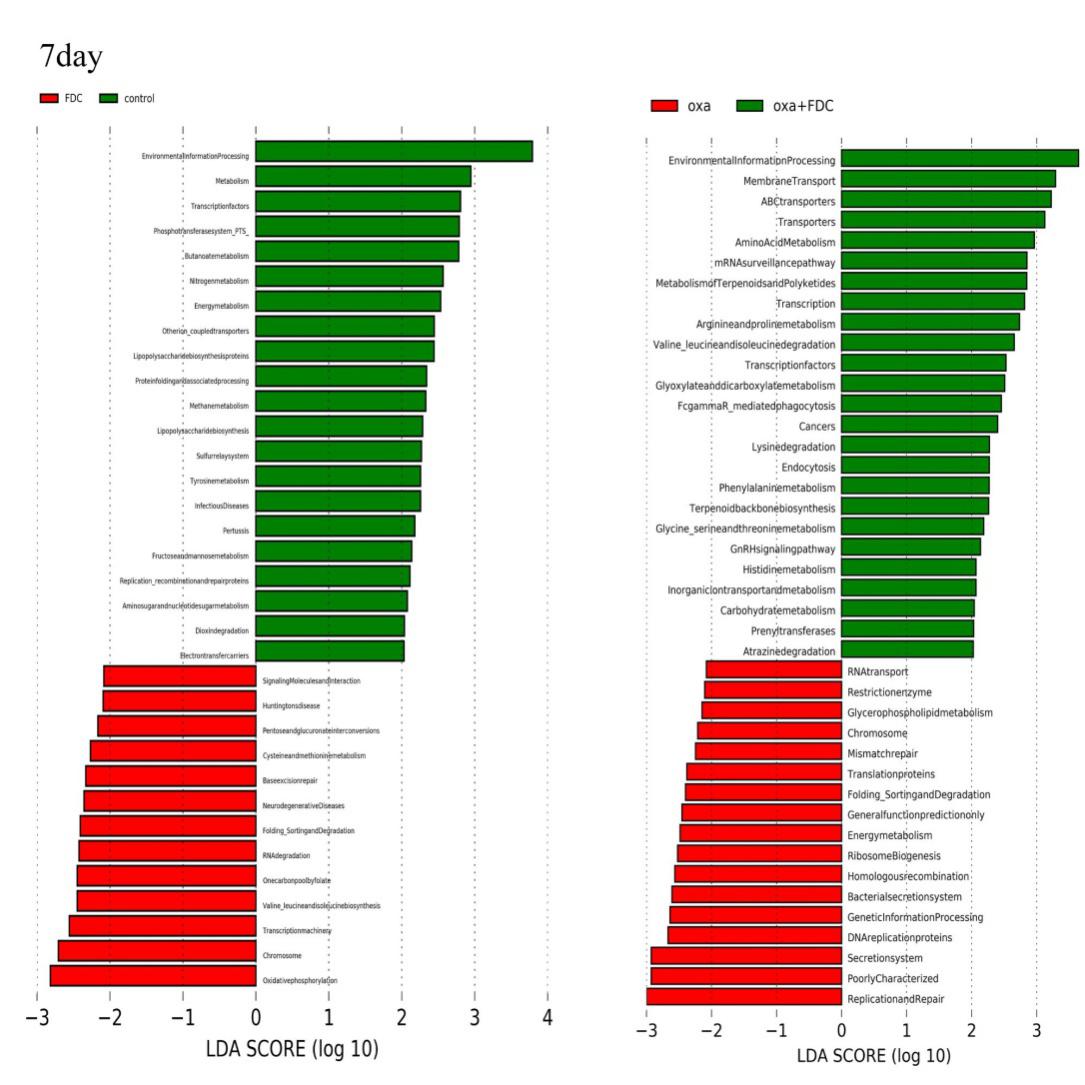


**Supplementary Figure 5.** KEGG analysis of genes differentially expressed in each group of tertiary metabolic pathways at 7 day.


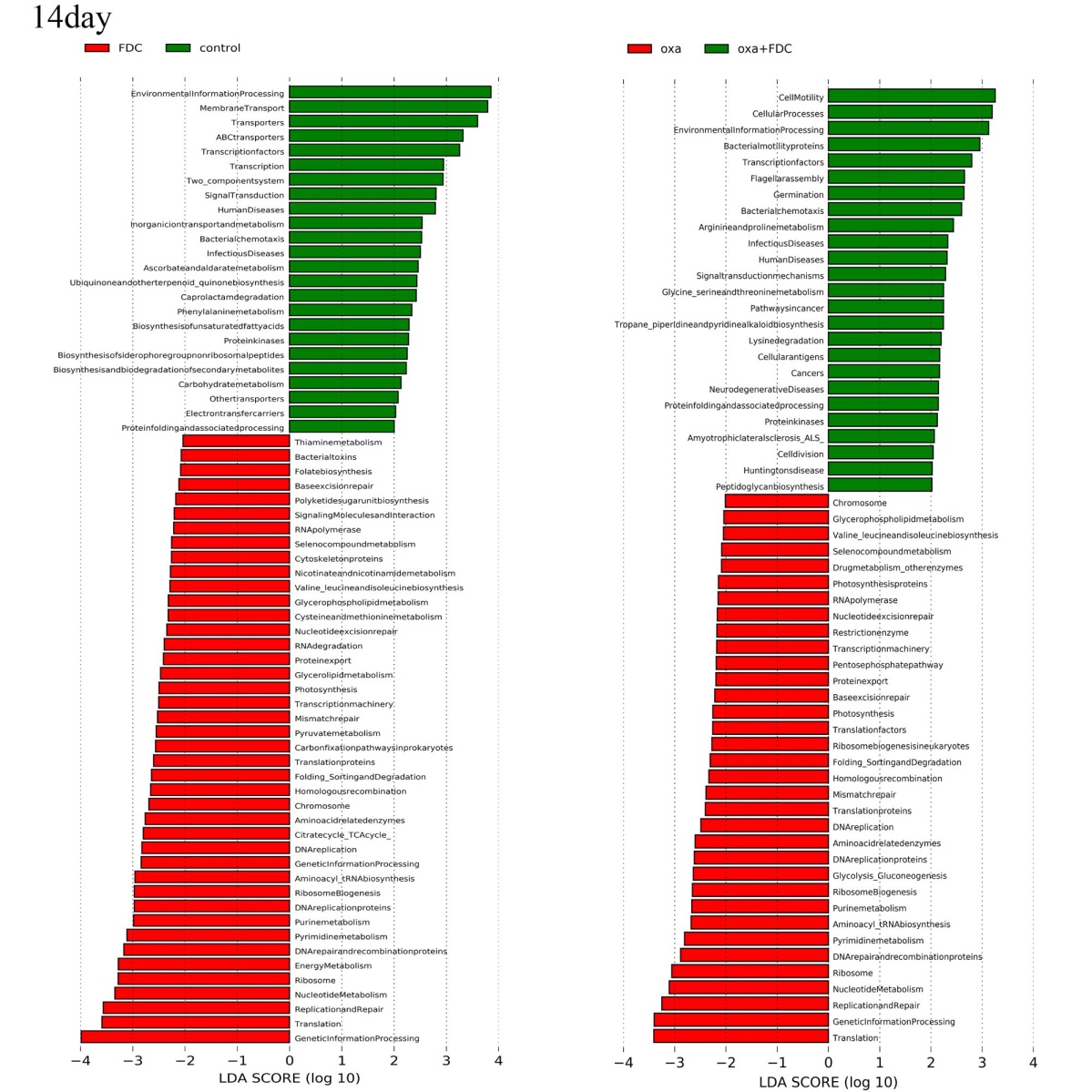


**Supplementary Figure 6.** KEGG analysis of genes differentially expressed in the tertiary metabolic pathway of each group at 14 day.
